# Pharmacological OGG1 inhibition decreases murine allergic airway inflammation

**DOI:** 10.3389/fphar.2022.999180

**Published:** 2022-10-17

**Authors:** Lloyd Tanner, Jesper Bergwik, Ravi K. V. Bhongir, Lang Pan, Caijuan Dong, Olov Wallner, Christina Kalderén, Thomas Helleday, Istvan Boldogh, Mikael Adner, Arne Egesten

**Affiliations:** ^1^ Department of Clinical Sciences Lund, Respiratory Medicine, Allergology, and Palliative Medicine, Lund University and Skåne University Hospital, Lund, Sweden; ^2^ Department of Microbiology and Immunology, University of Texas Medical Branch at Galveston, Galveston, United States; ^3^ Unit of Experimental Asthma and Allergy Research, Institute of Environmental Medicine (IMM), Karolinska Institutet, Stockholm, Sweden; ^4^ Science for Life Laboratory, Department of Oncology-Pathology, Karolinska Institutet, Stockholm, Sweden; ^5^ Oxcia AB, Stockholm, Sweden; ^6^ Weston Park Cancer Centre, Department of Oncology and Metabolism, University of Sheffield, Sheffield, United Kingdom

**Keywords:** allergic asthma, Ogg1, macrophage polarization, NF-κB, Th2 airway inflammation

## Abstract

**Background and aim:** Allergic asthma is a complex inflammatory disease involving type 2 innate lymphoid cells, type 2 T helper cells, macrophages, and eosinophils. The disease is characterized by wheezing, dyspnea, coughing, chest tightness and variable airflow limitation for which there is no cure and is symptomatically treated with inhaled corticosteroids and β2-agonists. Molecular mechanisms underlying its complex pathogenesis are not fully understood. However, 8-oxoguanine DNA glycosylase-1 (OGG1), a DNA repair protein may play a central role, as OGG1 deficiency decreases both innate and allergic inflammation.

**Methods:** Using a murine ovalbumin (OVA) model of allergic airway inflammation we assessed the utility of an inhibitor of OGG1 (TH5487) in this disease context. Cytokines and chemokines, promoting immune cell recruitment were measured using a 23-multiplex assay and Western blotting. Additionally, immune cell recruitment to bronchi was measured using flow cytometry. Histological analyses and immunofluorescent staining were used to confirm immune cell influx and goblet cell hyperplasia of the airways. A PCR array was used to assess asthma-related genes in murine lung tissue following TH5487 treatment. Finally, airway hyperresponsiveness was determined using *in vivo* lung function measurement.

**Results:** In this study, administration of TH5487 to mice with OVA-induced allergic airway inflammation significantly decreased goblet cell hyperplasia and mucus production. TH5487 treatment also decreased levels of activated NF-κB and expression of proinflammatory cytokines and chemokines resulting in significantly lower recruitment of eosinophils and other immune cells to the lungs. Gene expression profiling of asthma and allergy-related proteins after TH5487 treatment revealed differences in several important regulators, including down regulation of *Tnfrsf4, Arg1, Ccl12* and *Ccl11*, and upregulation of the negative regulator of type 2 inflammation, *Bcl6*. Furthermore, the gene *Clca1* was upregulated following TH5487 treatment, which should be explored further due to its ambiguous role in allergic asthma. In addition, the OVA-induced airway hyperresponsiveness was significantly reduced by TH5487 treatment.

**Conclusion:** Taken together, the data presented in this study suggest OGG1 as a clinically relevant pharmacological target for the treatment of allergic inflammation.

## Introduction

Asthma is a chronic inflammatory lung disease, affecting over 300 million people worldwide (The Global Asthma Report 2018). The disease is markedly heterogenous and complex, with both inherited susceptibility and environmental exposures playing important roles (Hay, 2017). Allergic asthma is characterized by specific IgE-secretion, accumulation of eosinophils in lung tissue, and increased mucus production, which leads to wheezing, dyspnea, coughing, chest tightness, and variable airflow limitation (Rodrigo et al., 2004). Primary treatment options for asthma focus on reductions in pulmonary inflammation as well as bronchodilation using inhaled corticosteroids, β2-agonists, and leukotriene receptor inhibitors (Reddel et al., 2021). However, a large proportion of asthma patients experience side effects, resulting in compromised treatment adherence (Cooper et al., 2015).

Type 2 driven airway inflammation is a key feature of allergic asthma, triggered by environmental antigens found in pollens, dust mites, fungi, and pet dander (Sporik et al., 1990; Suphioglu et al., 1992; Permaul et al., 2012). Release of type 2 cytokines, including IL-4, IL-5 and IL-13, results in an infiltration of several different immune cells mainly comprising eosinophils (Djukanovic et al., 1990; Holgate, 2012; Peters and Wenzel, 2020). Early events include epithelial cell activation of type 2 innate lymphoid cells (ILC2s) and, subsequently, IL-4 as a key cytokine for the conversion of naïve helper T cells into T_H_2 effector cells. Specialized subsets of T follicular helper cells (Tfh) produce IL-4 and are involved in B cell IgE production, which binds to the surface of mast cells causing degranulation (Li-Weber and Krammer, 2003). In models of allergy, Tfh cells acquire the ability to express IL-13, further promoting IgE production (Gowthaman et al., 2020). IgE-mediated degranulation of mast cells leads to release of proinflammatory mediators that cause bronchoconstriction and increase lung inflammation (Bradding et al., 2006; McBrien and Menzies-Gow, 2017). IL-5 is also an important regulator of eosinophils, which play a vital role in airway remodeling during allergic asthma, controlling differentiation, activation, and delaying apoptosis of immune cells (Takatsu and Nakajima, 2008).

Oxidative stress in the airways occurs during asthma due to release of reactive oxygen species (ROS) from activated inflammatory cells, in particular eosinophils (Kirkham and Rahman, 2006). One of the most abundant DNA lesions resulting from increased ROS is 8-hydroxy-2′-deoxyguanosine (8-oxoG) (Wu et al., 2004) and has been found to be increased in asthma patients (Zeyrek et al., 2009; Proklou et al., 2013). 8-oxoG lesions in chromatin of eukaryotic cells are predominantly repaired through base excision repair, which is initiated by 8-oxoG DNA glycosylase (OGG1) (David et al., 2007; Ba and Boldogh, 2018). Recent studies have also documented 8-oxoG formed in promoter-enhancer regions, with OGG1 shown to be a modulator of gene expression *via* the facilitation of transcription factor DNA occupancy (Li et al., 2012; Aguilera-Aguirre et al., 2017; Fleming and Burrows, 2017, 2020; Fleming et al., 2017; Ba and Boldogh, 2018; Tumurkhuu et al., 2020). *Ogg1* ^−/−^ and OGG1 siRNA murine studies (Klungland et al., 1999; Mabley et al., 2005; Bacsi et al., 2013) highlighted a reduced allergic inflammatory response after ovalbumin (OVA) challenge or ragweed pollen stimulation, respectively.

Recently, a small molecule inhibitor of OGG1, TH5487, was developed and shown to interfere with the binding of OGG1 to DNA in guanine-rich promotor regions, leading to reduced immune cell recruitment in a model of airway inflammation using lipopolysaccharide as a trigger (Visnes et al., 2018). In this study, we assessed the potential therapeutic use of TH5487 in a mouse model of allergen-induced airway inflammation. TH5487 treatment resulted in reduced immune cell recruitment to the lungs, lower levels of plasma IgE and OVA-specific IgE, decreased NF-κB activation in the lungs, decreased small air way mucus accumulation and less M2 macrophages in BALF and lung tissue. Together, these results suggest a potential role for OGG1 as a target to treat allergic asthma.

## Materials and methods

### Study design

The aim of this study was to evaluate the treatment potential of a small molecule inhibitor of OGG1 (TH5487), which prevents binding of OGG1 to oxidized DNA, against allergic asthma using an OVA-induced allergic inflammation mouse model. Pharmacological characteristics of TH5487 have been described elsewhere (Visnes et al., 2018). The mice were randomly divided into four groups, OVA (*n* = 9), OVA/TH5487 (*n* = 10), Vehicle (*n* = 10) and TH5487 (n = 10). When possible, downstream experiments were conducted with the investigator blinded to the sample groups. No animals were excluded as outliers.

### Ethical approval

Animal experiments were approved by the Malmö-Lund Animal Care Ethics Committee, ethical permit no. M3802-19 and Stockholm Animal Care Ethics Committee, ethical permit no. 3649-2019.

### Animals

**FIGURE 1:**
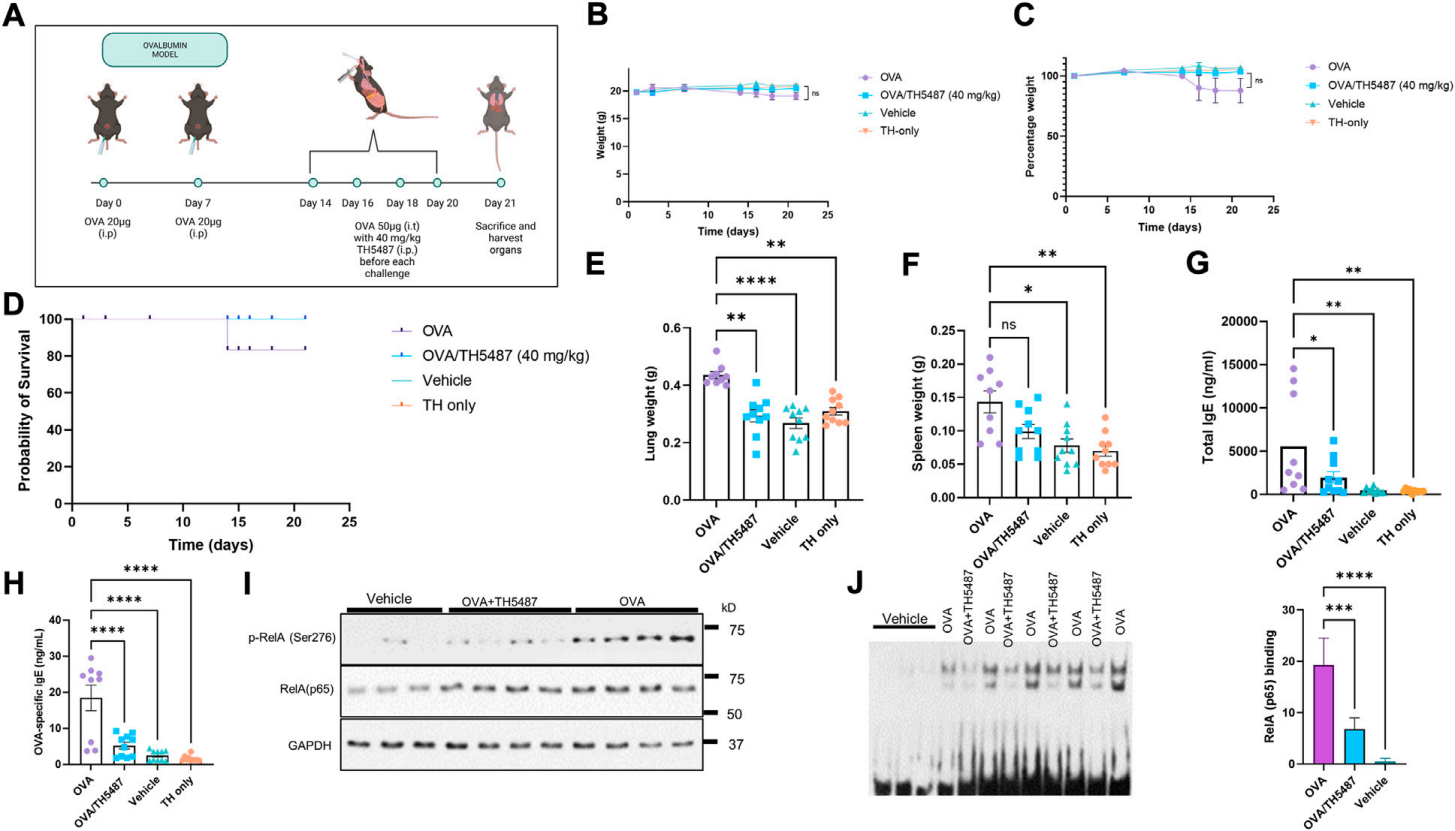
The effect of TH5487 treatment on survival, organ weights, NF-κB activation and IgE levels. **(A)** Sensitization and experimental schedule. All mice were sensitized with 20 µg ovalbumin (OVA) intraperitoneally at day 0 and 7. Mice in the OVA and OVA/TH5487 groups were challenged by intratracheal administration of 50 µg OVA at day 14, 16, 18 and 20. TH5487 treatment was performed by an intraperitoneal injection of 40 mg/kg before each challenge and all mice were sacrificed at day 21. **(B,C)** Total weights remained stable in all groups over the 21 days **(D)** Probability of survival showing insignificant differences between the groups. **(E)** Lung weight comparison between the groups showing a significant increase in the OVA group compared to OVA/TH5487, vehicle and TH5487 only groups. **(F)** Spleen weights showing a significant increase in the OVA group compared to vehicle and TH5487 only, and a trend towards an increase compared to OVA/TH5487. **(G)** Total and OVA-specific **(H)** IgE levels in plasma, which was increased in the OVA group and significantly reduced after TH5487 treatment. **(I)** Western blot analysis of RelA and phosphorylated RelA(NF-κB) (p-p65/RelA) showing a significant decrease in OVA/TH5487 compared to OVA. **(J)** Binding of NF-κB homo (p50-p50) and heterodimer (p50-p65) to consensus DNA sequences (5′-GGGRNYYYCC-3′) in extracts derived from individual lungs of control (vehicle), OVA/TH5487 and OVA-challenged mice. Statistical comparison between the groups were performed using a one-way ANOVA followed by a Dunnett’s post-hoc test (****p < 0.0001, ***p < 0.001, **p < 0.01, *p < 0.05).

Female C57BL/6J mice (Janvier, Le Genest-Saint-Isle, France) and male BALB/c (Envigo, Horst, NL) 8-10-week-old mice were housed in plastic cages with absorbent bedding material and were maintained for at least 2 weeks prior to initiation of experiments. The mice were kept in a controlled environment (temperature, light/dark cycle, food, and water *ad libitum*). Allergic airway inflammation was induced by sensitization with 20 µg OVA in alum (1:10) injected intraperitoneally at day 0 and 7 followed by challenges using intratracheal administration of 50 µg OVA at day 14, 16, 18 and 20 (Figure 1A). An intraperitoneal injection of TH5487 (40 mg/kg) was performed prior to each challenge and mice were sacrificed at day 21. The mice were randomly allocated into four groups: OVA sensitized (vehicle), OVA sensitized + OVA challenged (OVA), OVA sensitized + OVA challenged + TH5487 (OVA/TH5487), and OVA sensitized + TH5487 (TH only). The lung function experiments were performed in BALB/C mice as this strain develops a stronger airway hyperresponsiveness (AHR) than C57BL/6 mice (Swedin et al., 2010).

### Blood collection

Collection of blood in tubes containing 0.5 M EDTA was performed by cardiac puncture. The tubes were centrifuged at 1,000 x *g* for 10 min and the supernatants were kept for later analysis.

### Lung tissue collection

The left lung from each mouse was collected in Histofix (Histolab, Göteborg, Sweden) and transferred to buffered paraformaldehyde solution (4%). The right lung was submerged in RNAlater solution (Thermo Fisher Scientific, Waltham, MA) and stored at −20°C. After thawing, sample aliquots were homogenized in tissue protein extraction reagent (T-PER) solution (Thermo Fisher Scientific) with proteinase inhibitor (Pefabloc, SC; Sigma-Aldrich, Saint Louis, MI) at a concentration of 1 mM. Following homogenization, samples were centrifuged at 9,000 x *g* for 10 min at 4°C and the supernatants were collected for later analysis. The remaining sample was used for RNA extraction (see below).

### Bronchoalveolar lavage fluid collection

BAL was performed using a total volume of 1 ml PBS with 100 µM EDTA. The BALF was kept on ice and aliquoted for flow cytometry, cytospin differential counts and multiplex cytokine analysis. Cytospin samples were stained with modified Giemsa-Wright stain (Sigma-Aldrich) or used for immunofluorescence staining (see below).

### SDS-PAGE and western blotting

Total protein concentrations of lung homogenate lysates were analyzed with a Pierce BCA Protein Assay Kit (Thermo Fisher Scientific). SDS-PAGE was performed using Mini-PROTEAN^®^ Precast Mini PAGE Gels (Bio-Rad, Hercules, CA). Trans-Blot Turbo Mini 0.2 µM PVDF Transfer Packs (Bio-Rad) were used for transferring of proteins to the PVDF membranes. Membranes were blocked for 3 h at RT and incubated with primary antibodies (rabbit anti-mouse NF-κB(RelA/p65/) (sc-8008; Santa Cruz Biotechnology, Santa Cruz, CA), rabbit anti-mouse phospho (p)-RelA/p65(NF-κB) (Ser276; A1953; Abcam), mouse anti-mouse arginase-1 (ab239731), rabbit anti-mouse CD206 (ab64693) and rabbit anti-mouse GAPDH (14C10; Cell Signaling; 1:500) in blocking buffer. After washing with PBS-Tween, membranes were incubated with secondary antibodies (Alexa Fluor 488-conjugated goat anti rabbit/mouse (Invitrogen, Carlsbad, CA)) for 1 h RT. Imaging of blots was preformed using a ChemiDoc system (Bio-Rad) followed by quantification with densitometry normalized to GAPDH.

### Total IgE ELISA

Total plasma IgE levels were determined using a IgE Mouse ELISA Kit (Invitrogen, Waltham, MA). Plasma samples diluted 1:500, and IgE standard (0.137–100 ng/ml) were pipetted into a 96-well plate and incubated for 2.5 h (RT) with gentle shaking. Wells were washed 4 times with wash buffer followed by addition of biotin conjugate to all wells. After 1 h incubation at RT with gentle shaking, the wells were washed 4 times and Streptavidin-HRP solution was added to all wells. The wash was repeated and TMB substrate was added following incubation for 30 min at RT with gentle shaking. The reaction was stopped by adding Stop Solution and absorbance was read at 450 nm using a VICTOR 1420 Multilabel plate reader (PerkinElmer, Waltham, MA). A standard curve was generated (Sigmoidal, 4 PL) and used to calculate IgE levels in the samples.

### OVA-specific IgE ELISA

OVA-specific IgE levels were measured using a LEGEND MAX™ Mouse OVA Specific IgE ELISA Kit (BioLegend, San Diego, CA). The plate was washed 4 times with wash buffer followed by addition of Matrix A to standard wells and Assay Buffer to sample wells. Standard (20-0.313 ng/ml) and samples (diluted 1:2) were added to the plate and incubated for 2 h with shaking at 200 rpm. After washing the wells 4 times, Mouse Detection Antibody solution was added to each well followed by incubation for 1 h with shaking at 200 rpm. Wells were washed 4 times and Avidin-HRP D solution was added to each well and incubated for 30 min while shaking. The wells were washed 5 times, with soaking of the wells for 30 s to 1 min between each wash, before adding Substrate Solution F followed by incubation for 15 min in the dark. The reaction was stopped with Stop Solution and the absorbance was read at 450 nm using a VICTOR 1420 Multilabel plate reader (PerkinElmer). Using the absorbance of the standard, a linear standard curve was generated to calculate IgE levels in the samples.

### MUC5AC ELISA

The standards and BAL fluid samples were added to plates pre-coated with a MUC5AC antibody (MyBioSource Cat # MBS2507150). Thereafter, biotinylated antibody specific for MUC5AC and Avidin-Horseradish Peroxidase (HRP) conjugate was added to each well. Plates were washed and substrate added to each well. Reactions were terminated by the addition of a sulphuric acid solution. The optical density was measured spectrophotometrically at a wavelength of 450 nm using Synergy H1 BIO-TEK Instruments (Winooski, VT).

### Immunostaining of lung sections

Fixated lung tissue sections underwent antigen retrieval (pH 9 buffer) using a Dako PT Link pre-treatment module (Agilent, Santa Clara, CA). Samples were washed with PBS and blocked with Dako protein block (Agilent) for 10 min, followed by incubation with primary antibodies overnight (mouse anti-mouse arginase-1 (ab239731), mouse anti-mouse MUC5ac (MA5-12178)). Samples were incubated with secondary antibodies, Alexa Fluor 594 goat anti mouse (Abcam, Cambridge, United Kingdom). Glass cover slips were mounted with DAPI- containing fluoroshield (Abcam). Images were visualized using a Nikon Confocal Microscope (Nikon, Tokyo, Japan) and fluorescence was quantified using ImageJ software (https://imagej.nih.gov).

### Immunostaining of BALF samples

Cytospin preparations of BALF cells were blocked with Dako protein block (Agilent) for 10 min and incubated with primary antibodies (mouse anti-mouse arginase-1 (ab239731)) for 1 h RT. Secondary antibodies, Alexa Fluor 594 goat anti-mouse (Abcam), was added to the samples, followed by incubation for 30 min at RT. Slides were mounted with cover slips and DAPI-containing fluoroshield (Abcam). Images were visualized using a Nikon Confocal Microscope and fluorescence was quantified using ImageJ software.

### Real-time PCR array

Total mRNA was extracted from lung tissue submerged in RNAlater using an RNeasy Mini Kit (Qiagen, Hilden, Germany) according to the protocol from the manufacturer. RNA concentrations were determined using a NanoDrop ND1000 (Saveen Werner, Limhamn, Malmö). Equal amounts of RNA were pooled from several animals in each group (OVA/TH5487 *n* = 5, OVA *n* = 4). cDNA was synthesized with an iScript Advanced cDNA Synthesis Kit (Bio-Rad) and mixed with RT^2^ SYBR^®^ Green ROX™ qPCR Mastermix. A volume of 25 µL of the reaction mixture was added to each well of a RT^2^ Profiler™ PCR Array Mouse Allergy & Asthma PAMM-067ZA plate. The RT-PCR reaction was performed using a QuantStudio™ 7 Flex system (Thermo Fisher Scientific) and data analysis was performed using the manufacturer’s web-based software (https://geneglobe.qiagen.com/analyze). Normalization of gene expression was performed using the following house-keeping genes: *B2m*, *Actb*, *Gusb*, *Gapdh* and *Hsp90ab1*.

### Electrophoretic mobility shift assay

Snap frozen lungs were homogenized, and nuclear extracts were obtained using the CelLyticTM NuCLEARTM Extraction Kit (Millipore-Sigma). Protein concentrations were determined by Pierce BCA Protein Assay Kit (Thermo Fisher Scientific). EMSA assays were performed as described previously (Pan et al., 2016; Hao et al., 2018). Briefly, biotin-labeled probes (20 fmol; Sense: 5′-TTCCCTGGTCCCCGGGCTTTTCCAGACATCG-3′Anti-sense:5′-biotin CGATGTCTGGAA AAG​CCC​GGG​GAC​CAG​GGA​A-3′) were mixed with 2 µg extract in binding buffer (10 mM Tris-Cl (pH 8.0), 10 mM NaCl, 1 mM DTT, 1 mM EDTA, 1 mg/ml BSA and 0.1 μg/μl Poly [d (I-C). Protein-DNA complexes were resolved on 6% nondenaturing polyacrylamide gels (Invitrogen) in 0.25 × TBE buffer (100V for 1.5 h). Images were visualized using Amersham Imager 680 (Global Life Sciences Solutions, Marlborough, MA). Band intensities were quantified using ImageJ v1.51 (NIH, Bethesda, MD).

### Flow cytometry

A BD Accuri 6 (BD, Franklin Lakes, NJ) was used for the flow cytometry experiments. The cells were washed in stain buffer 1x (BD554656) followed by incubation with Lyse Fix 1x (BD558049 (5x)). After fixing, the samples were washed with stain buffer and separated into two equal samples. The first sample was incubated with either rat anti-mouse CD45 (BD553080), anti-CD11b (BD553312), anti-CD11c (BD558079) and anti-Ly6G (BD551461), with the second sample incubated with rat anti-mouse CD45 (BD553080), anti-CD11b (BD553312), anti-CD11c (BD558079) and anti-SiglecF (BD562680).

### Bioplex cytokine analysis

A Bio-Plex Pro mouse cytokine assay (23-Plex Group I; BioRad) using a Luminex-xMAP/Bio-Plex 200 System was used to quantify multiple cytokines in BALF, plasma and lung homogenate. Analysis was performed using Bio-Plex Manager 6.2 software (Bio-Rad). The detection limits were as follows: CCL11 (Eotaxin) (21372.02-1.15 pg/ml), GCSF (124018.4-6.97 pg/ml), GMCSF (1,161.99-3.73), IFN-γ (14994.64-0.72 pg/ml), IL-1α (10337.5-0.63 pg/ml), IL-1β (28913.54-1.57 pg/ml), IL-2 (22304.34-1.21 pg/ml), IL-3 (7,639.21-0.47 pg/ml), IL-4 (6,334.86-0.36 pg/ml), IL-5 (12950.39-0.76 pg/ml), IL-6 (11370.16-0.66 pg/ml), IL-9 (2,580.93-2.46 pg/ml), IL-10 (76949.87-4.09 pg/ml), IL-12p40 (323094.58-17.38 pg/ml), IL-12p70 (79308.46-19.51 pg/ml), IL-13 (257172.3-53.85 pg/ml), IL-17 (8,355.61-0.5 pg/ml), KC (23377.88-1.3 pg/ml), MCP-1 (223776.6-45.04 pg/ml), MIP-1α (14038.07-0.58 pg/ml), MIP-1β (928.18-2.39 pg/ml), RANTES (4,721.74-4.42 pg/ml), and TNF-α (73020.1-4.61 pg/ml). Correction for protein concentration was done using a Pierce ™ BCA Protein Assay Kit (Thermo Fischer Scientific).

### H&E and PAS staining of lung sections

Right lungs were fixed in Histofix (Histolab), and paraffin embedded. Sections (3 µm) were cut with a microtome and placed on glass slides (Superfrost Plus; Thermo Fisher Scientific). Deparaffinization was performed using serial baths of xylene and ethanol. Staining was completed using Mayer hematoxylin and 0.2% eosin (Histolab) or Periodic Acid Schiff (PAS) Stain Kit (Mucin Stain) (Abcam). Imaging of the slides was performed using an Olympus BX60F microscope with an SC50 camera (Olympus, Tokyo, Japan).

### Real-time PCR

Total mRNA was extracted from lung tissue kept in RNAlater (stored at −20°C) using a RNeasy Mini Kit (Qiagen, Valencia, CA) according to the manufacturer’s protocol. RNA concentrations were determined with a NanoDrop ND1000 (Saveen Werner, Malmö, Sweden). RNA was converted into cDNA (1 µg) using an iScript Advanced cDNA Synthesis Kit (Bio-Rad). Expression of target genes was measured using TaqMan™ Fast Advanced Master Mix with TaqMan™ probes listed in Supp. Table 1tbl1, and the reactions were run on a CFX Connect Real-Time System in 96-well plates. Water samples were included to confirm non-specific PCR reactions. ΔCt values were calculated by normalization to house-keeping gene succinate dehydrogenase complex, subunit A (*Sdha*). To obtain ΔΔCt values, ΔCt values from the treatment groups were divided by ΔCt values from the control group. Fold change was calculated by 2^−ΔΔCt^.

### Measurement of airway hyperresponsiveness

On day 21 of the OVA-challenge protocol, AHR was induced by administration of methacholine (MCh; Sigma-Aldrich) in mice anaesthetized with ketamine hydrochloride (75 mg/kg, Ketaminol^®^ Vet., Intervet, Stockholm, Sweden) and medetomidine hydrochloride (1 mg/kg, Cepetor®Vet., VETMEDIC, Stockholm, Sweden). Methacholine was delivered by aerosol administration *via* a nebuliser (Scireq; Montreal, Que., Canada), at doses ranging from 0 to 12.5 mg/ml after an initial dose of saline alone. The AHR was measured with a small animal ventilator (FlexiVent; Scireq), as previously described. Dynamic and central resistance (R and Rn, respectively), central airway compliance (C), peripheral tissue damping (G) and tissue elastance (H) were recorded. Newtonian resistance, Rn, is a predictive measure of resistance in the central airways, with tissue damping reflecting energy dissipation in the lung tissue, and tissue elastance indicating tissue stiffness.

### Statistical analysis

Analysis of differences between three or more groups was calculated using one-way ANOVA with Dunnett’s post hoc test. Statistical testing was performed using GraphPad Prism 9.3.1 (350) (GraphPad Software, San Diego, CA) and the statistical significance was defined as *p* < 0.05. The results are displayed as mean ± SEM.

## Results

### TH5487 treatment decreases levels of plasma IgE and activated NF-κB

Allergic airway inflammation was induced by ovalbumin (OVA)-sensitization and challenge (Figure 1A). An intraperitoneal injection of TH5487 (40 mg/kg) was performed prior to each challenge. No significant differences in weight loss or probability of survival were seen between the groups (Figures 1B–D). Lung weights were significantly increased in the OVA group compared to the TH5487 treated mice (Figure 1E) and a similar trend was seen for the spleen weight, without reaching statistical significance (Figure 1F). Total IgE and OVA-specific IgE levels in plasma were both significantly decreased in the OVA/TH5487 group compared to the OVA group (Figures 1G,H). Activation of the NF-κB signaling pathway plays a central role in allergic inflammation of both patient-derived samples and in OVA-sensitized mice (Poynter et al., 2002; Gagliardo et al., 2003). Western blot analysis of NF-κB displayed increases in the phosphorylated catalytic subunit (p-RelA (p65) the mammalian homolog of the V-Rel avian reticuloendotheliosis viral oncogene A) in the OVA-challenged group, whereas treatment with TH5487 resulted in no significant changes in levels of p-RelA/p65(NF-κB) (Figure 1I). To support these observations, EMSAs were performed, showing increased binding to probes of homo (p50-p50) and heterodimeric (pRelA-p50) complexes of NF-κB in lung extracts from OVA-challenged mice. In lung extracts of OVA challenged TH5487-treated mice there were decreased levels of both p50-p50 and p50-p65 bound to the probes (Figure 1J).

### Goblet cell hyperplasia and airway mucin production are decreased following TH5487 treatment

**FIGURE 2:**
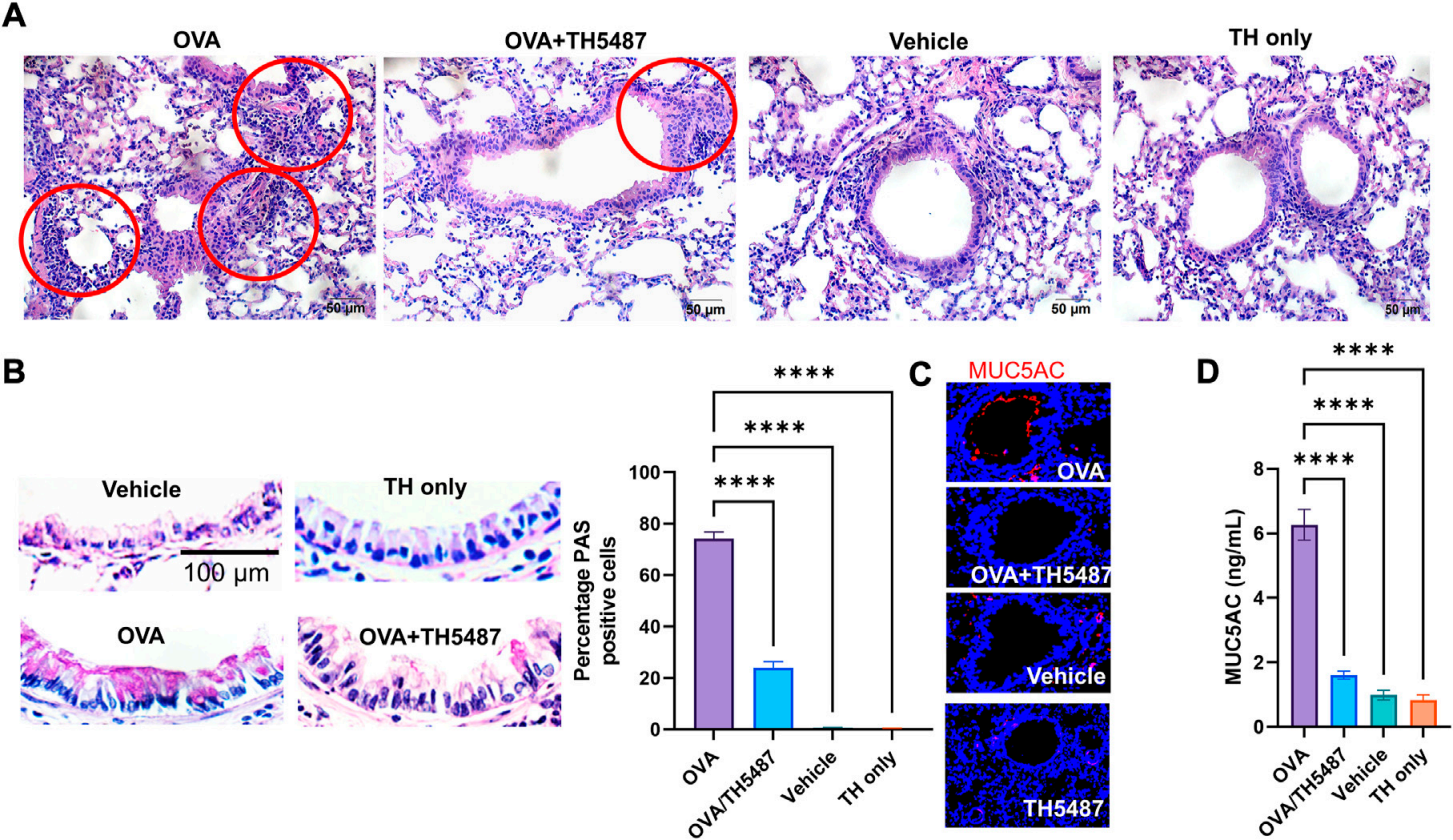
H&E, PAS and immunofluorescence staining of murine lung sections. Mouse lungs were harvested, formalin fixed, sectioned, and stained with haemotoxylin and eosin (H&E), periodic acid schiff (PAS) or fluorescent antibodies. **(A)** Representative images of H&E-stained lung sections from OVA-challenged mice with and without TH5487 treatment. Inflammatory infiltrates surrounding bronchi/bronchioles (red circles); OVA: lung section of ovalbumin-challenged mouse; OVA + TH5487: lung section of TH5487-treated OVA challenged mouse; Vehicle: control vehicle alone; TH only: TH5487 treatment alone. Scale bar: 50 µm. **(B)** TH5487 decreases mucin-containing cells (Goblet cells) as shown by PAS staining (magenta). Upper left panel, a representative image from unchallenged (vehicle), TH5487 only (upper right panel), OVA (lower left panel) and OVA/TH5487 (lower right panel) challenged/treated lungs. Magenta-colored epithelial cells are positive for mucin. Percentage of mucin producing cells were enumerated in epithelium of bronchioles from 3 sections of each lung by two independent investigators. Percentage of mucin positive cells were calculated and graphically depicted. Scale bar: 100 µm. **(C)** Murine lung sections were stained with MUC5AC antibody (red) to determine mucin production, with **(D)** MUC5AC ELISA conducted on homogenized murine lung tissues. Statistical comparison between the groups were performed using a one-way ANOVA followed by a Dunnett’s post-hoc test (****p < 0.0001).

Lungs of sensitized animals challenged with OVA, OVA/TH5487, vehicle or TH5487 only were sectioned and stained with H&E or PAS stains. Histological analyses of H&E-stained sections showed increased cellularity, especially around primary and secondary bronchi and bronchioles in OVA-challenged groups. Significantly decreased levels of accumulated inflammatory cells were seen in lungs of TH5487 treated animals (Figure 2A and Supplementary Figures S1–3). After PAS staining, bronchiolar mucosal epithelium in lungs of OVA-challenged animals exhibited increased goblet cell hyperplasia (Figure 2B). TH5487-treatment of OVA-challenged mice significantly (*p < 0.001) decreased PAS positive cells compared with OVA alone (Figure 2B), while vehicle and TH5487 displayed no PAS positive cells (Figure 2B). Figure 2C shows immunochemical staining of MUC5AC (upper panel) in OVA, which was decreased by TH5487 treatment of OVA sensitized/challenged animals. The histological observations are supported by ELISA conducted on BALF samples, showing significantly decreased MUC5AC levels in the OVA/TH5487, compared to that of OVA alone group (Figure 2D).

### Immune cell recruitment to the lung is mitigated by treatment with TH5487

**FIGURE 3:**
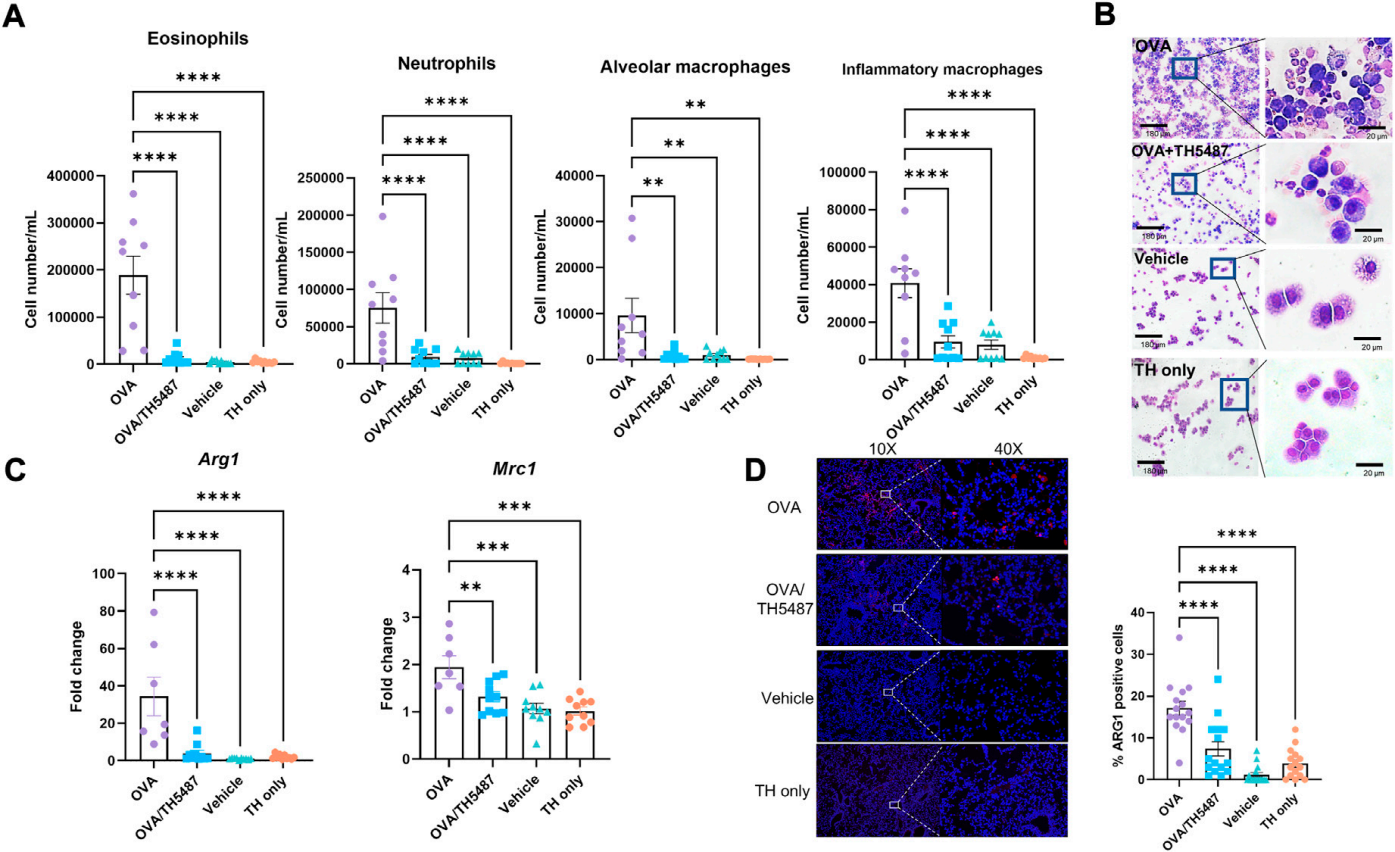
Immune cell recruitment measured in murine BALF. **(A)** A significant increase of eosinophils, inflammatory macrophages, alveolar macrophages, and neutrophils was seen in the OVA-challenged group. TH5487 treatment significantly reduced recruitment of all cell types to the lung. **(B)** Giemsa-Wright-stained cytospins of BALF samples showing an increase in the number of immune cells in the OVA group compared to OVA/TH5487 vehicle and TH5487 only groups, respectively. **(C)** RT-PCR measured expression of *Mrc1*, which encodes CD206, and *Arg1* is significantly reduced in the OVA/TH5487 group compared to the OVA group. **(D)** Murine lung sections stained with ARG1 antibodies revealing decreases in ARG1 positive cells in the OVA/TH5487 group compared to OVA. Statistical comparisons were performed using a one-way ANOVA followed by a Dunnett’s post-hoc test (****p < 0.0001, ***p < 0.001, **p < 0.01).

In allergic asthma, recruitment of immune cells to the airways results from an increased release of cytokines/chemokines from the airway epithelium and from resident immune cells (Velazquez and Teran, 2011). The effect of TH5487 on immune cell recruitment to the lungs was investigated by performing flow cytometry on BALF from OVA-challenged mice. Large increases in the number of eosinophils, inflammatory macrophages, alveolar macrophages, and neutrophils were seen in the OVA challenged mice compared to the control mice (Figures 3A,B; Supplementarty Figure S4). Administration of TH5487 resulted in significant reductions of all immune cells, with almost no detection of eosinophils and macrophages. BALF samples stained with Giemsa-Wright revealed an increased number of immune cells in the OVA challenged mice compared to the OVA/TH5487, vehicle and TH5487 only treated mice (Figure 3B).

Type 2 inflammation, which is a key feature of allergic asthma, has been shown to induce polarization of macrophages towards an M2 phenotype (Girodet et al., 2016; Nie et al., 2017). Gene expression of the M2 markers *Mrc1*, which encodes the surface receptor CD206, and *Arg1* in the lungs of the mice was shown to be significantly reduced in the OVA/TH5487 mice compared to the OVA mice (Figure 3C). Western blot analysis using lung homogenates revealed a significant increase in ARG1 in the OVA challenged mice compared to the OVA/TH5487, vehicle and TH5487 only treated mice (Supplementarty Figure S5A). A trend towards a similar difference was seen for CD206. Immunofluorescence staining of mouse BALF samples with ARG1 antibodies showed a significant increase in ARG1 positive cells in the OVA group compared to OVA/TH5487 (Supplementarty Figure S5B). Furthermore, ARG1 immunofluorescence staining of lung sections further confirmed a significant increase of ARG1 positive cells in the OVA group compared to the OVA/TH5487 group (Figure 3D).

### Administration of TH5487 reduces murine cytokine levels in BALF, lung and plasma

**FIGURE 4:**
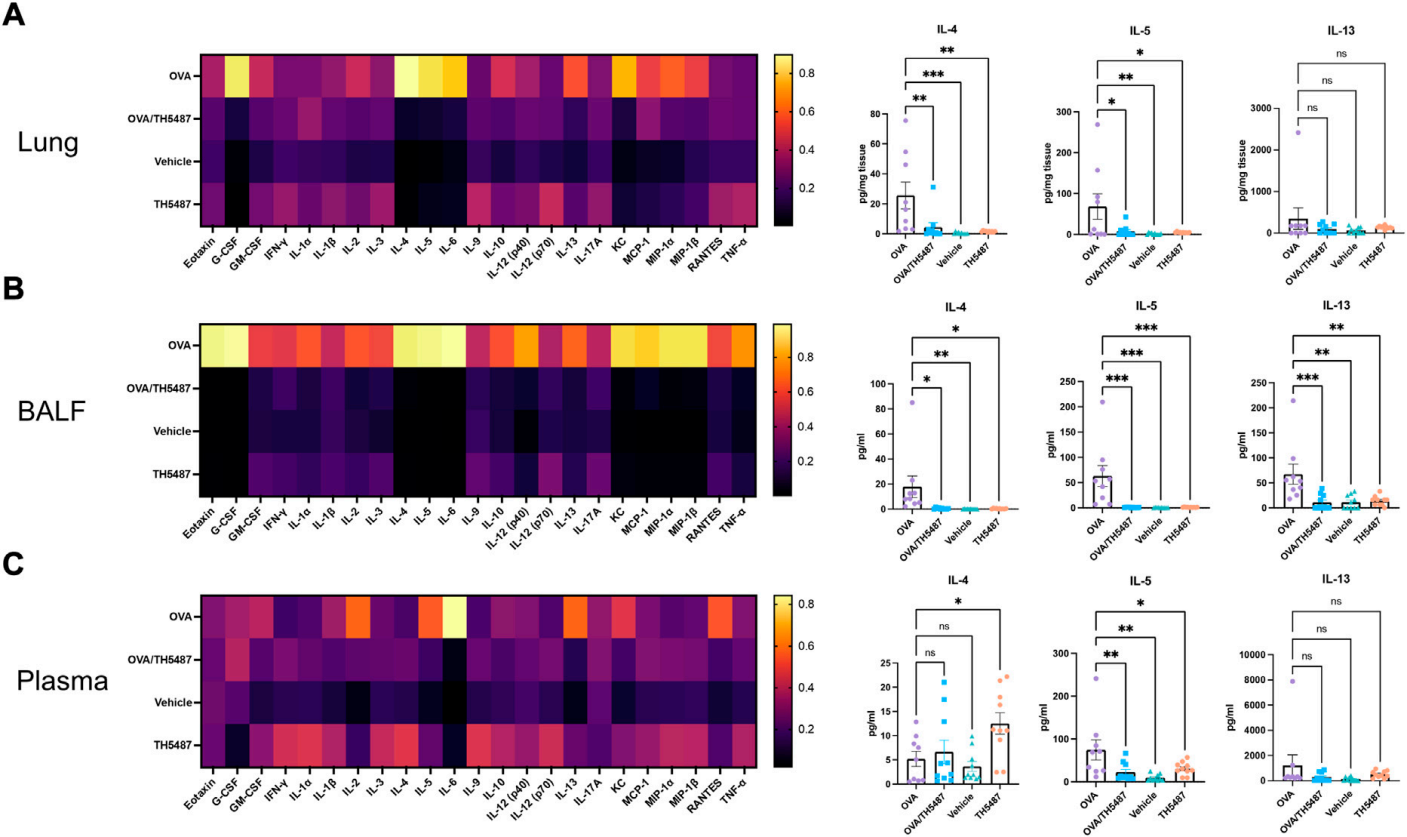
Murine cytokine levels in lung, BALF and plasma. **(A–C)** Heatmaps displaying differences in cytokine levels in lung homogenate, BALF and plasma (yellow indicates high value; black indicates low value). The key T_H_2 cytokines IL-4, IL-5 and IL-13 are shown as individual graphs including statistical comparisons. Differences in cytokine levels were compared using a one-way ANOVA followed by a Dunnett’s post-hoc test (***p < 0.001, **p < 0.01, *p < 0.05).

Pro-inflammatory cytokines were measured in lung homogenate, BALF and plasma using a 23-cytokine multiplex assay. Noticeable increases in several cytokines were seen in the OVA group, indicating increased allergic inflammation in the lungs of these mice (Figures 4A–C, left and right panels and Supplementary Figures S6–8). Treatment with TH5487 resulted in a significant decrease in a majority of cytokines in the BALF. Moreover, a considerable decrease of the type 2 cytokines IL-4, IL-5 and IL-13 was shown in the TH5487 treated mice, suggesting a reduced type 2 response. Eotaxin (CCL11) levels in the BALF were decreased after TH5487 treatment, which partly explains the decreased recruitment of eosinophils to the lungs. In the OVA treated mice, a significant increase was seen in monocyte chemotactic and activating factor (CCL2) in the BALF (*p* < 0.0001), indicating an increased recruitment of monocytes and macrophages to the lung. However, treatment with TH5487 resulted in a significant reduction of CCL2 (*p* < 0.0001) to levels nearly equal to the vehicle and TH5487 only groups, further supporting decreased immune cell recruitment to the lung. Unexpectedly, higher levels of IL-4 were found in plasma from the TH5487 only group (*p* = 0.0277).

### Gene expression profiling reveals decreases in expression of key type 2 response genes by TH5487 administration

**FIGURE 5:**
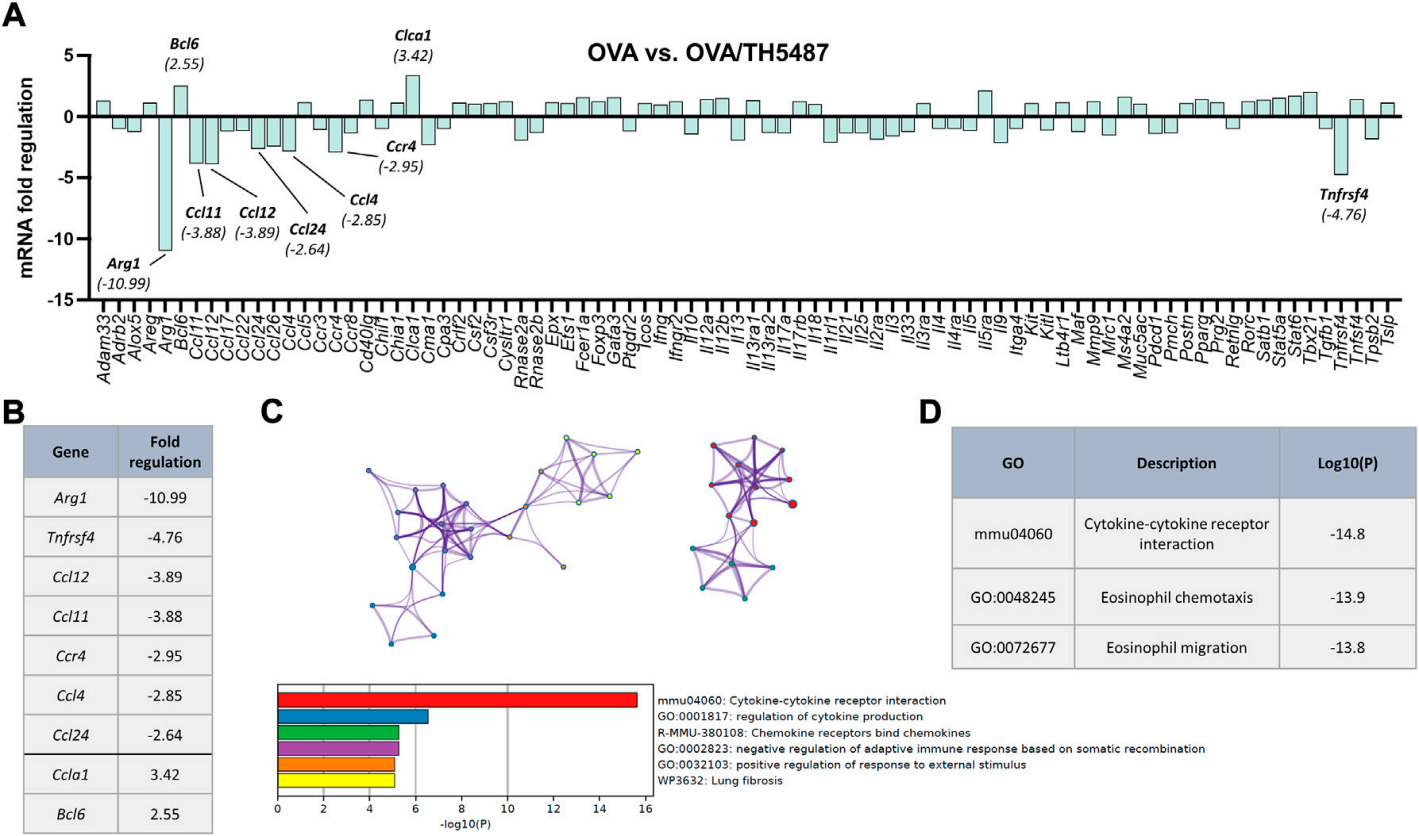
Asthma and allergy related gene expression profiling of lung tissue. **(A)** Asthma and allergy gene array comparison between OVA and OVA/TH5487. Equal amounts of mRNA from each mouse were pooled (OVA *n* = 4, OVA/TH5487 *n* = 5). Individual genes with a difference in fold regulation of >2.5 are highlighted in the graph. **(B)** List of genes with a fold regulation of >2.5. **(C)** Metascape protein network analysis displaying key proteins (>Log2.5) and the associated gene-ontology terms (color-coded). **(D)** Key gene-ontology terms displaying significantly downregulated Log10P values following treatment with TH5487.

Using a RT^2^ Profiler™ PCR Array Mouse Allergy & Asthma panel, the expression of 84 allergy and asthma related genes in the lung was quantified (Figure 5A). Comparison between the OVA and OVA/TH5487 groups revealed a difference in mRNA fold regulation of several genes (Figure 5B). The largest decrease in expression (−10.99 fold) was seen in the macrophage M2-marker *Arg1*, indicating a decreased number of M2 macrophages in the lung. Furthermore, the expression of *Tnfrsf4*, which is involved in activation of T-cells, was decreased after TH5487 treatment (−4.76 fold). A decrease was also seen in *Ccl11* (eotaxin), an eosinophil-specific chemokine (−3.88 fold). Two genes were shown to be upregulated, i.e., *Bcl6* (2.55 fold) and *Clca1* (3.42). Interestingly, BCL6 reduces type 2 responses through transcriptional repression of several key cytokines and chemokines (Arima et al., 2008). CLCA1 is a regulator of mucus production in goblet cells, but studies describing its role in allergic asthma have been contradictory (Nakanishi et al., 2001; Robichaud et al., 2005). Using Metascape, a resource for analysis of system-level datasets (Zhou et al., 2019), gene ontology (GO) terms associated with the results from the gene expression profiling were generated (Figures 5C,D). The most significant GO terms were cytokine-cytokine receptor interaction (Log10P (−14.8)), eosinophil chemotaxis (Log10P (−13.9)) and eosinophil migration (Log10P (−13.8)).

### Improved lung function in OVA-challenged TH5487-treated animals

**FIGURE 6:**
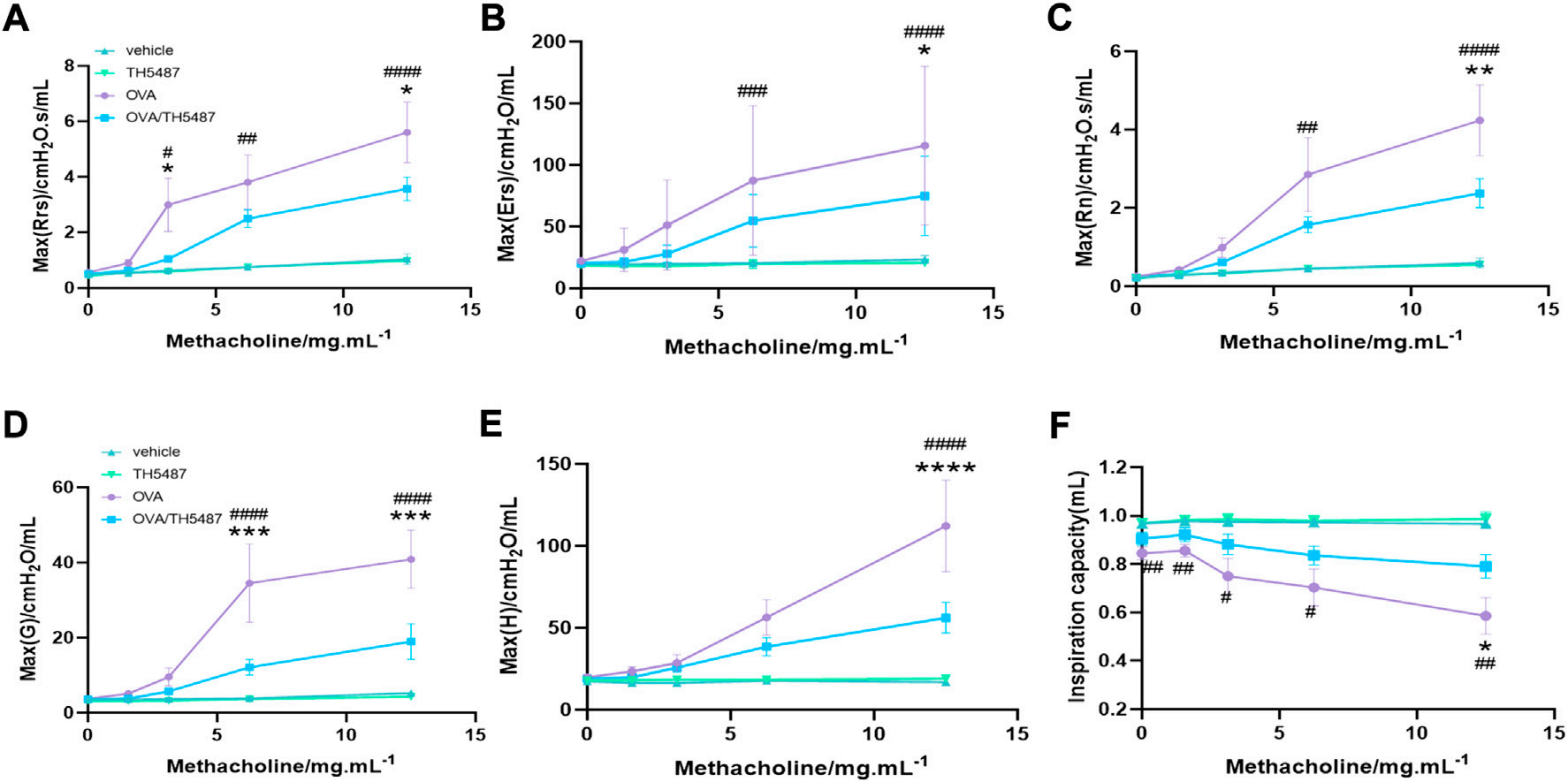
TH5487-mediated differences in airway function in OVA-challenged mice. Airway hyperresponsiveness (AHR) was measured with a small animal ventilator (FlexiVent; Scireq), as described in Materials and Methods. **(A–C)** Dynamic, elastic, and central resistance (Rrs, Ers, and Rn, respectively), peripheral tissue damping **(D)**, tissue elastance **(E)**, and central airway compliance **(F)** were recorded. The results are expressed as mean ± SEM (*n* = 4–9). Vehicle only (vehicle), TH5487 only (TH5487), ovalbumin-challenged (OVA), and ovalbumin-challenged/TH5487-treated (OVA/TH5487). Statistical comparisons were performed using a one-way ANOVA followed by a Dunnett’s post-hoc test (****p < 0.0001, ***p < 0.001, **p < 0.01, *p < 0.05, vs. respective OVA/TH5487, ####*p* < 0.0001, ###*p* < 0.001, ##*p* < 0.01, #*p* < 0.05 vs. respective vehicle).

To assess AHR, OVA-challenged mice were administered methacholine (MCh), a substance known to induce smooth muscle constriction similar to hyperreactivity of the allergic asthmatic response seen clinically. Our results showed significantly elevated levels in OVA-challenged mice (*p* < 0.05) of dynamic (Rrs) and central resistance (Rn) at 12.5 mg/ml MCh compared to the vehicle and TH5487-treated animals (Figures 6A–C). TH5487/OVA mice displayed decreased tissue dampening and decreased tissue elastance compared to OVA-challenged mice (*p* < 0.001 and *p* < 0.0001, respectively; Figures 6D,E). Subsequently, inspiration capacity was significantly decreased in OVA-challenged mice compared to TH5487-treated animals (*p* < 0.05; Figure 6F).

## Discussion

Allergic asthma is a highly complex and heterogeneous inflammatory disease involving multiple cell types and tissues (Holgate et al., 2015). Molecular mechanisms underlying its pathogenesis are not well understood, however, it is clear that ROS are important in allergic inflammation and oxidatively modified molecules including intrahelical 8-oxoG (Wu et al., 2000; MacPherson et al., 2001; Nadeem et al., 2003; Sahiner et al., 2011). Recent studies have identified 8-oxoG in promoter regions appearing as epitranscriptomic-like marks, with OGG1 DNA repair coupled to transcriptional regulation of chemokines and cytokines (Ba et al., 2014; Ba and Boldogh, 2018; Hao et al., 2018, 2020). Therefore, we tested whether pharmacological targeting of OGG1 decreased allergic immune responses in an OVA-challenged mouse model. We showed that the small molecule TH5487, which selectively inhibits the binding of OGG1 to oxidatively modified guanines, significantly decreased transcription from pro-inflammatory genes. Additionally, TH5487 treatment reduced the recruitment of eosinophils and other inflammatory cells as well as decreasing mucin production and bronchial hyperreactivity, displaying promising results as potential treatment against allergic inflammation and asthma.

Allergic asthma is a disease characterized by dysregulated expression of type 2 cytokines in the airways, resulting in increased recruitment of inflammatory cells such as eosinophils and macrophages. Eosinophils are significant contributors to oxidative stress through the release of reactive oxygen species (ROS), including nitrogen oxide (NO)-derived oxidants that cause damage of proteins through modification of tyrosine residues (Wu et al., 2000; MacPherson et al., 2001). CCL11 (eotaxin-1) plays a central role in the recruitment of eosinophils and is primarily produced by the epithelium in response to allergen exposure within the airways. However, during an allergen challenge the primary source of CCL11 is switched to macrophages (Rothenberg, 1999). In asthmatic patients and OVA-sensitized mice, macrophages are polarized towards an M2 phentoype (Girodet et al., 2016; Nie et al., 2017), suggesting that these cells are an important source of CCL11 during allergic asthma. In this study, we found a reduced expression of *Ccl11* in lung tissue and lower CCL11 levels in the BALF after treatment with TH5487, which could be explained by a reduction in M2 macrophages. Indeed, reductions in the expression of the two M2 markers *Arg1* and *Mrc1* were seen in lung tissue of mice treated with TH5487. This was further established by immunofluorescent staining of ARG1 in cytospins from BALF samples and lung sections, where TH5487 administration resulted in less immunoreactivity. M2 macrophages produce several different factors, including the anti-inflammatory cytokine IL-10. Studies in mice lacking expression of IL-10 have shown an increased survival of type 2 cells, leading to an increased expression of IL-5 which exacerbates pulmonary inflammation through an influx of eosinophils (Yang et al., 2000; Coomes et al., 2017). Moreover, M2 macrophages further exacerbate the type 2 response by releasing IL-13, CCL17, CCL18, CCL22, CCL24 (eotaxin-2), and CCL26 (eotaxin-3), thereby increasing eosinophil chemotaxis (Siddiqui et al., 2013). Consequently, a reduction of M2 macrophages in allergic asthma would decrease the production of type 2 cytokines and reduce eosinophil recruitment to the lungs.

Gene expression profiling of lung tissue from OVA and OVA/TH5487 mice respectively, revealed differences in mRNA fold changes of several genes. Two genes were upregulated more than 2.5-fold, including *Bcl6*, which has been shown to negatively regulate type 2 responses and macrophage related chemokines (Arima et al., 2008). Knockout of *Bcl6* in mice results in eosinophilic inflammation due to an overproduction of type 2 cytokines (Dent et al., 1997). The ability of BCL6 to reduce the type 2 response is due to its ability to regulate the expression of several genes. BCL6 decreases the expression of IL-5 in T-cells by binding to a silencing element of the IL-5 gene (Arima et al., 2002). BCL6 also suppresses the expression of IL-6 and CCL2 in macrophages (Toney et al., 2000; Yu et al., 2005) as well as MIP-1α and NF-κB in lymphocytes (Shaffer et al., 2000; Li et al., 2005). However, several studies have shown IL-6 signaling is involved with CCL2 production which promotes the prolonged recruitment of monocytes and macrophages, immune cells which sustain inflammation within the lung (Gabay, 2006; Lee et al., 2008; Farahi et al., 2017; Esnault et al., 2021). Additionally, *Tnfrsf* 4 was downregulated by TH5487, a gene known to attenuate T cell-mediated responses (Herrick and Bottomly, 2003; Ward-Kavanagh et al., 2016; Martín-Orozco et al., 2017). More specifically, CD4^+^ T cell responsiveness to allergen exposure correlates well with asthma diagnosis, with associated increases in type 2 cytokines, increased total mucins, and increased MUC5AC in the airways of allergic asthma patients (Cho et al., 2016). Whilst, T cell recruitment was not measured during this study, type 2 cytokines and MUC5AC were both decreased by TH5487 treatment. Finally, *Bcl6* ^*−/−*^ mice have increased amounts of IgE due to immunoglobulin class switching in B cells (Harris et al., 1999). In our study, administration of TH5487 resulted in significantly lower concentrations of IL-5, IL-6, CCL2 and MIP-1α in BALF, decreased phosphorylation of NF-κB in the lungs and a reduction in total IgE and OVA-specific IgE, which is consistent with the increased expression of *Bcl6* after TH5487 treatment.

The effectiveness of TH5487 stems from the inhibition of OGG1, which has pleiotropic roles in gene expression by binding to intrahelical 8-oxoG located in regulatory regions and facilitates transcription factor DNA occupancy (Schreck et al., 1990; Ghosh and Mitchell, 1999; Pan et al., 2017; Hao et al., 2018). OGG1 creates specific DNA structural changes that facilitate NF-κB (and other transcription factors) recognition and binding to its consensus motif (Schreck et al., 1990; Ghosh and Mitchell, 1999; Bruner et al., 2000; Pan et al., 2017). In addition, OGG1-mediated incision of DNA as part of base excision repair is also linked to modulation of gene expression (Pastukh et al., 2015; An et al., 2016; Zhu et al., 2018; Fleming et al., 2019). Excision of 8-oxoG by OGG1 generates apurinic sites in gene regulatory regions, particularly in promoters of inflammatory genes (An et al., 2016; Zhu et al., 2018; Fleming et al., 2019). Studies using *Ogg1* knockout animals and biochemical approaches have shown distinct roles for OGG1 in gene expression in a stimuli- and context-dependent manner (Perillo et al., 2008; Sampath et al., 2012; Hao et al., 2018; Sampath and Lloyd, 2019; Simon et al., 2020). This phenomenon is also true for NF-κB of which activity is dependent upon activation pathway(s), posttranslational modifications, and combinatorial effects of subunits (Cartwright et al., 2016). Importantly, treatment with TH5487 during OVA-induced airway hyperresponsiveness was significantly attenuated, indicative of an effect on the airway reactivity that is highly relevant for human asthma-related symptoms.

A limitation of this study is whether treatment of allergic asthma with TH5487 is feasible in humans, for which further studies are needed. Another important limitation of this study is the administration route of the drug, which is injected intraperitoneally in the mice. Due to the acute nature of allergic asthma a rapid and more directed route would be optimal, such as an inhaler. No evident changes in general murine health status were seen in this study, but more long-term monitoring of potential side effects is required before initiating clinical trials. However, previous studies on TH5487 (Visnes et al., 2018, 2020; Baquero et al., 2021) and *Ogg1* ^*−/−*^ mice (Li et al., 2012) showed no adverse health effects in short term experimentation. Conversely, long term studies on *Ogg1* ^*−/−*^ mice showed increased risk for tumorigenesis (Sakumi et al., 2003), a result which should be studied further in TH5487 long term trials.

Collectively, our findings display OGG1 inhibition by TH5487 as a novel, potent pharmacological approach to treat allergic asthma. TH5487 inhibition of proinflammatory genes prevents type 2 driven downstream activation of immune cells, resulting in reduced NF-κB activation, decreased immune cell recruitment to the lungs, lowered levels of IgE and OVA-specific IgE in plasma, reduced mucus production in the small airways, improved airway function and finally, decreased M2 macrophage populations in BALF and lung tissue. These data support further development of TH5487 and other OGG1-inhibitors as templates for novel drugs against allergic asthma.

## Data Availability

The original contributions presented in the study are included in the article/Supplementary Material, further inquiries can be directed to the corresponding author.
