# Supplementary material for: Pharmacological OGG1 inhibition decreases murine allergic airway inflammation
Source: Front Pharmacol. 2022 Oct 17;13:999180. doi: 10.3389/fphar.2022.999180 (PMC9619105; doi:10.3389/fphar.2022.999180)
Supplement: Supplementary file 1 [file Table1.DOCX]

**Pharmacological OGG1 inhibition decreases murine allergic airway inflammation**

**Running title:** OGG1-inhibition alleviates allergic asthma

**Lloyd Tanner^1*^, Jesper Bergwik^1^, Ravi KV Bhongir^1^, Lang Pan^2^, Caijuan Dong^3^, Olov Wallner^4^, Christina Kalderén^4,5^ Thomas Helleday^4,5,6^, Istvan Boldogh^2^, Mikael Adner^3^, Arne Egesten^1^**

^1^Respiratory Medicine, Allergology, and Palliative Medicine, Department of Clinical Sciences Lund, Lund University and Skåne University Hospital, Lund, Sweden.

^2^Department of Microbiology and Immunology, University of Texas Medical Branch at Galveston, Galveston, TX77555, USA

^3^Unit of Experimental Asthma and Allergy Research, Institute of Environmental Medicine (IMM), Karolinska Institutet, Stockholm, Sweden.

^4^Science for Life Laboratory, Department of Oncology-Pathology, Karolinska Institutet, SE-171 76 Stockholm, Sweden.

^5^Oxcia AB, Norrbackagatan 70C, SE-113 34 Stockholm

^6^Weston Park Cancer Centre, Department of Oncology and Metabolism, University of Sheffield, Sheffield S10 2RX, UK.

***Correspondence:**Dr. Lloyd Tanner, BMC B14, SE-221 84 Lund, Sweden.

[lloyd.tanner@med.lu.se](mailto:lloyd.tanner@med.lu.se)

Keywords: Allergic asthma, OGG1 inhibitor, macrophage polarization, NF-κB, T_H_2 cytokines

**Supplementary Materials**

**
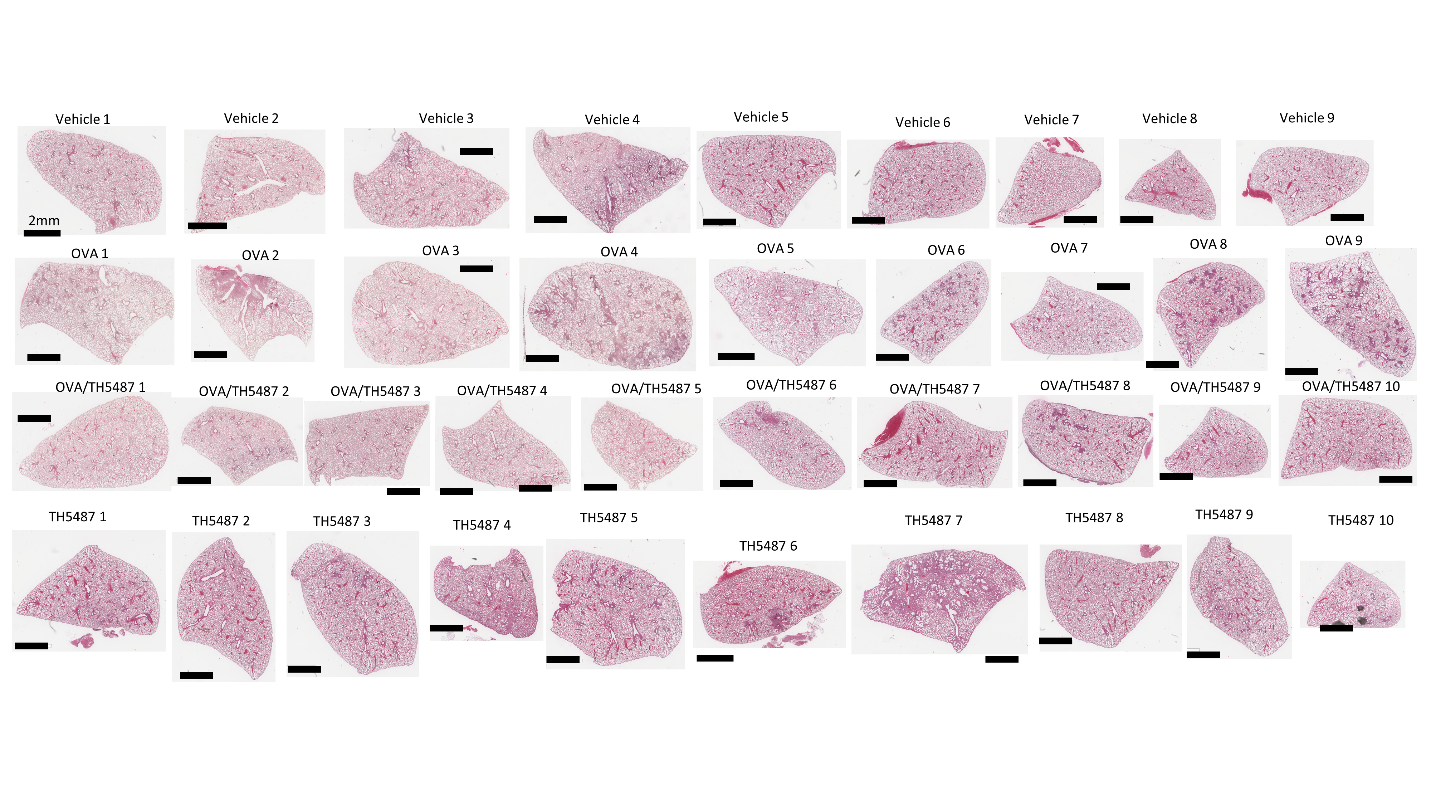
**

**Supplementary figure 1: Whole lung scans from murine lungs stained with H&E.** Mice were allocated into four groups: OVA sensitized (vehicle), OVA sensitized (TH group), OVA sensitized + OVA challenged (OVA), and OVA sensitized + OVA challenged + TH5487 (OVA/TH5487). Scale bar = 2 mm.


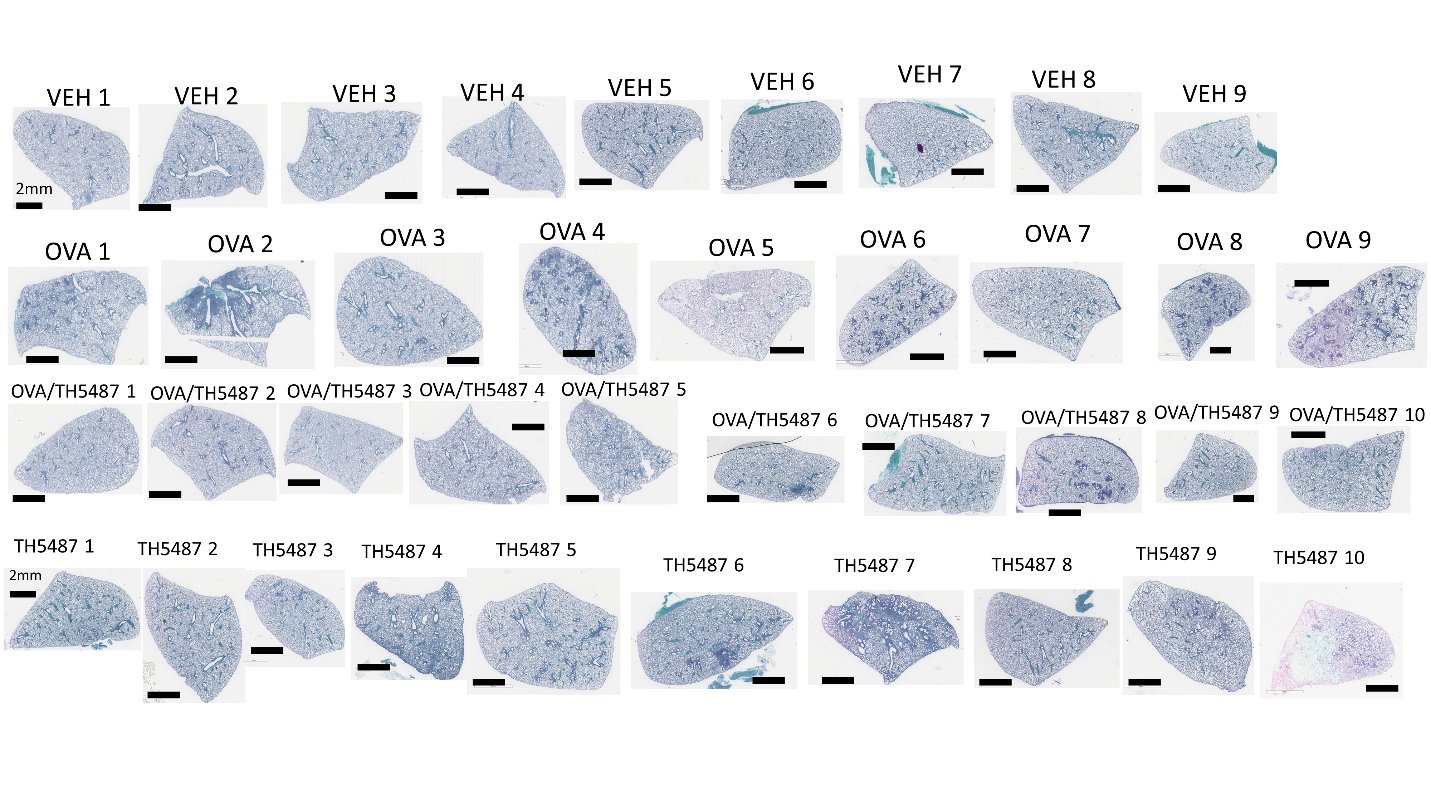


**Supplementary figure 2: Whole lung scans from mice showing periodic acid-Schiff (PAS) staining.** Mice were allocated into four groups: OVA sensitized (vehicle), OVA sensitized (TH group), OVA sensitized + OVA challenged (OVA), and OVA sensitized + OVA challenged + TH5487 (OVA/TH5487). Scale bar = 2 mm.


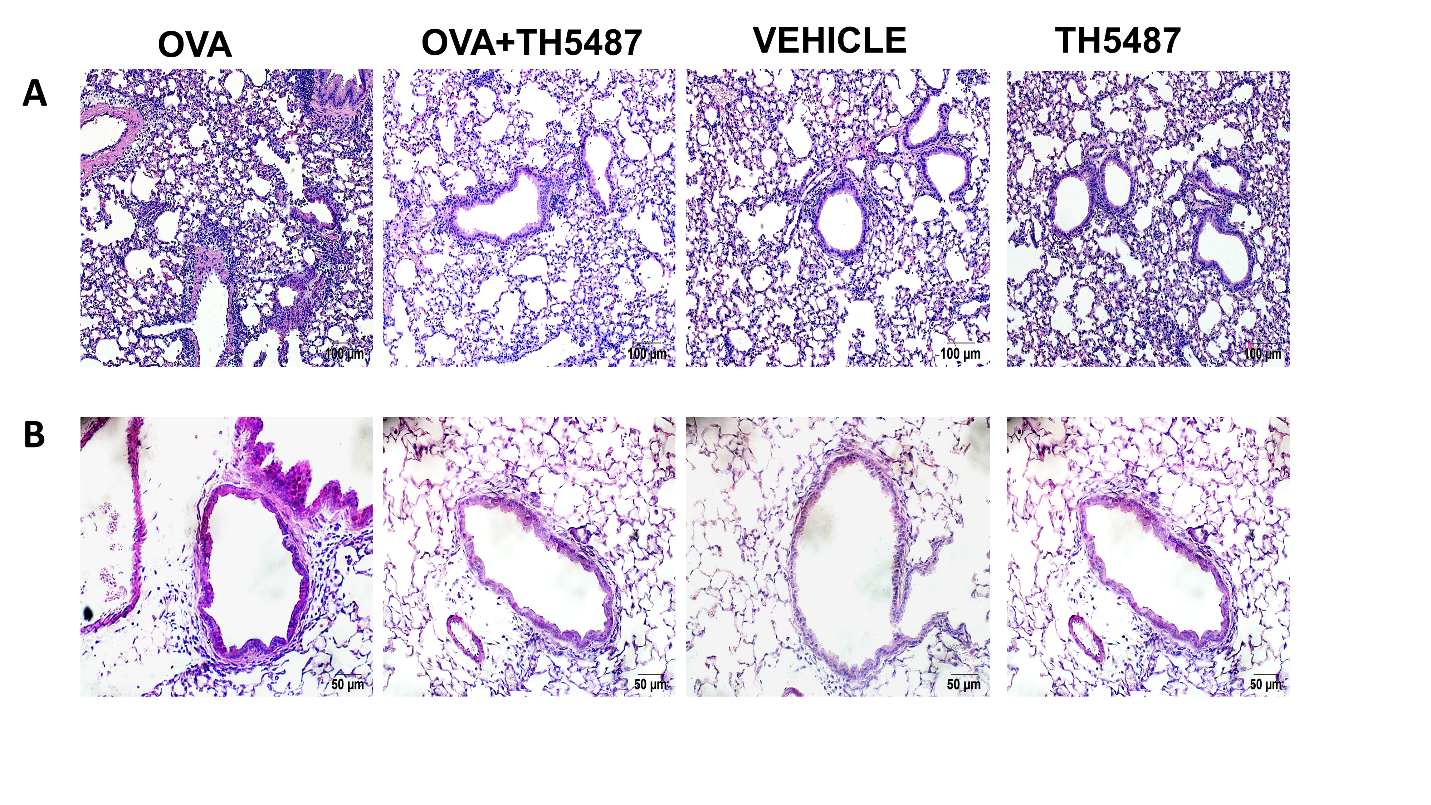


**Supplementary Figure 3: Representative images from mouse lungs showing (A) inflammatory cell influx and (B) periodic acid-Schiff (PAS) staining following OVA challenge/TH5487 treatment.** Scale bars = 100 μm and 50 μm.

**
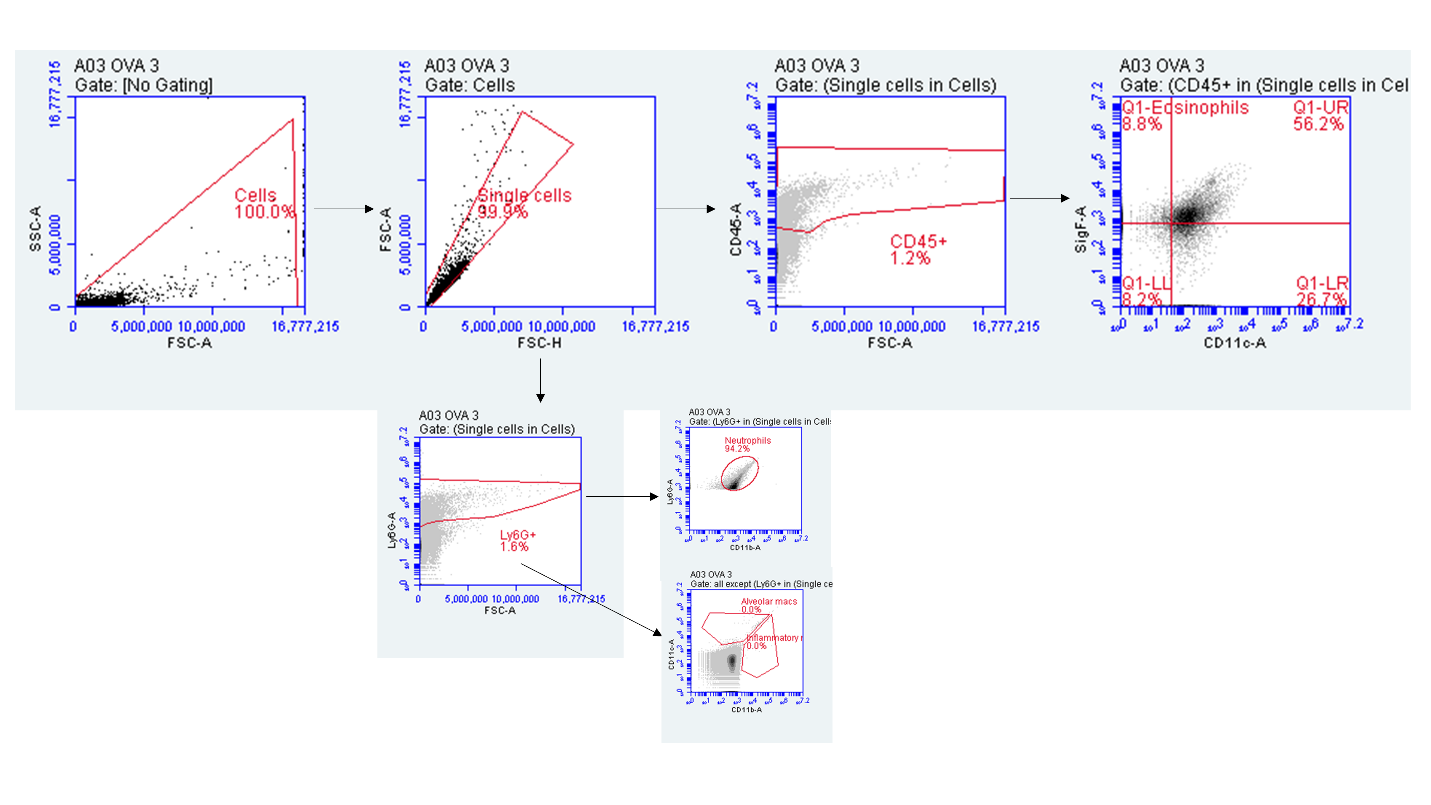
**

**Supplementary Figure 4: Representative gating strategy for flow cytometry analysis.** Eosinophils (CD45+/SigF+/CD11c-), neutrophils (CD45+/Ly6G+/CD11b+), and macrophage subsets (inflammatory macrophages: Ly6G-/CD11c+/CD11b- and alveolar macrophages: Ly6G-/CD11c-/CD11b+) were gated accordingly.

###
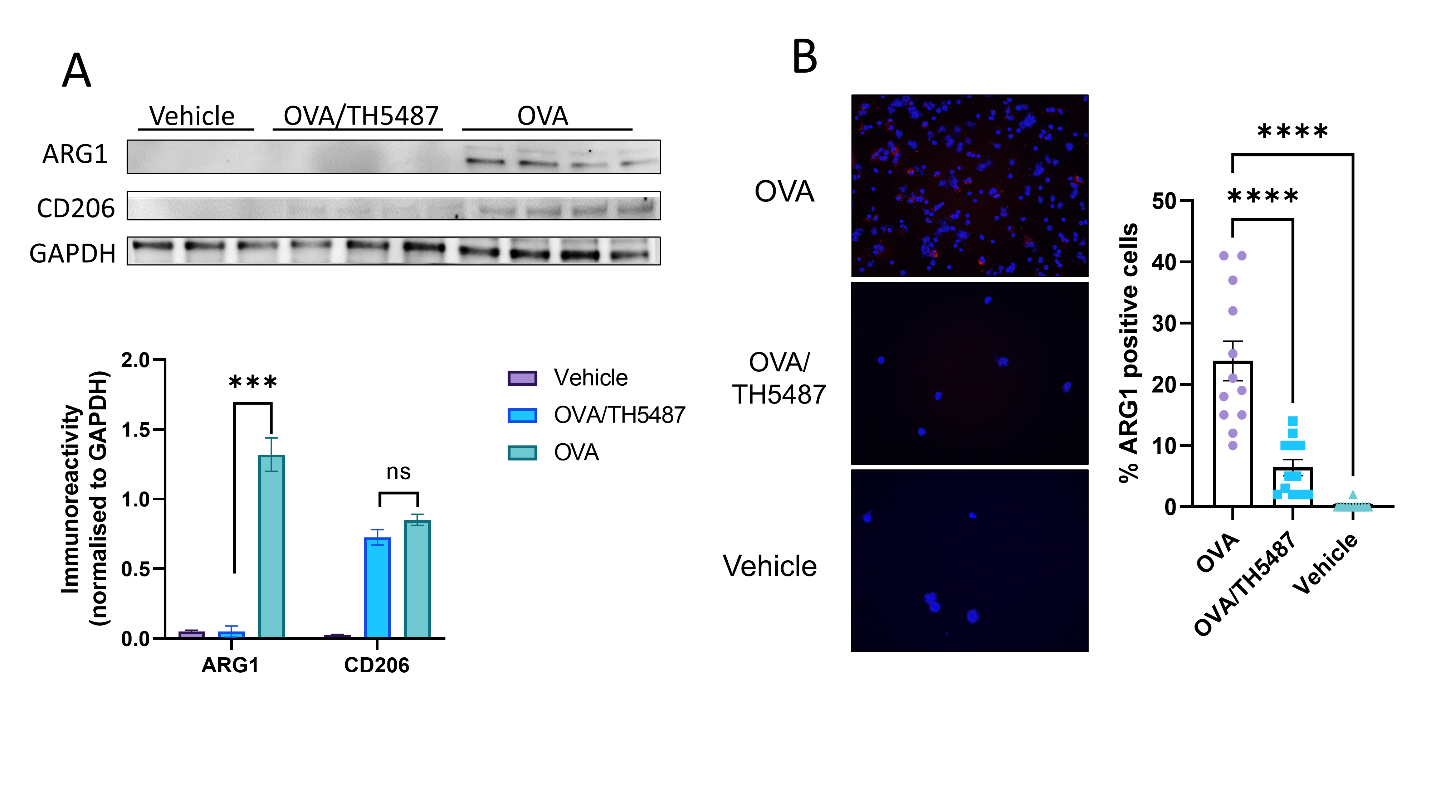


### **Supplementary Figure 5: TH5487-driven differences in macrophage M2 polarization in mouse lung and BALF.** (A) Western blot analysis of ARG1 and CD206 showing significant reduction of ARG1 after TH5487 treatment compared to the OVA group. (B) Cytospins from BALF samples stained with Arg1 antibodies showing an increase in Arg1 positive cell in the OVA group, which is significantly reduced by TH5487 treatment. Statistical comparisons were performed using a one-way ANOVA followed by a Dunnett’s post-hoc test (*****P*<0.0001, ****P*<0.001, ***P*<0.01, **P*<0.05).

**Supplementary figure 6:** Individual graphs of all cytokines measured in lung homogenate. Statistical comparison between groups was performed using a one-way ANOVA with Dunnett’s post-hoc test (*****P*<0.0001, ****P*<0.001, ***P*<0.01, **P*<0.05).


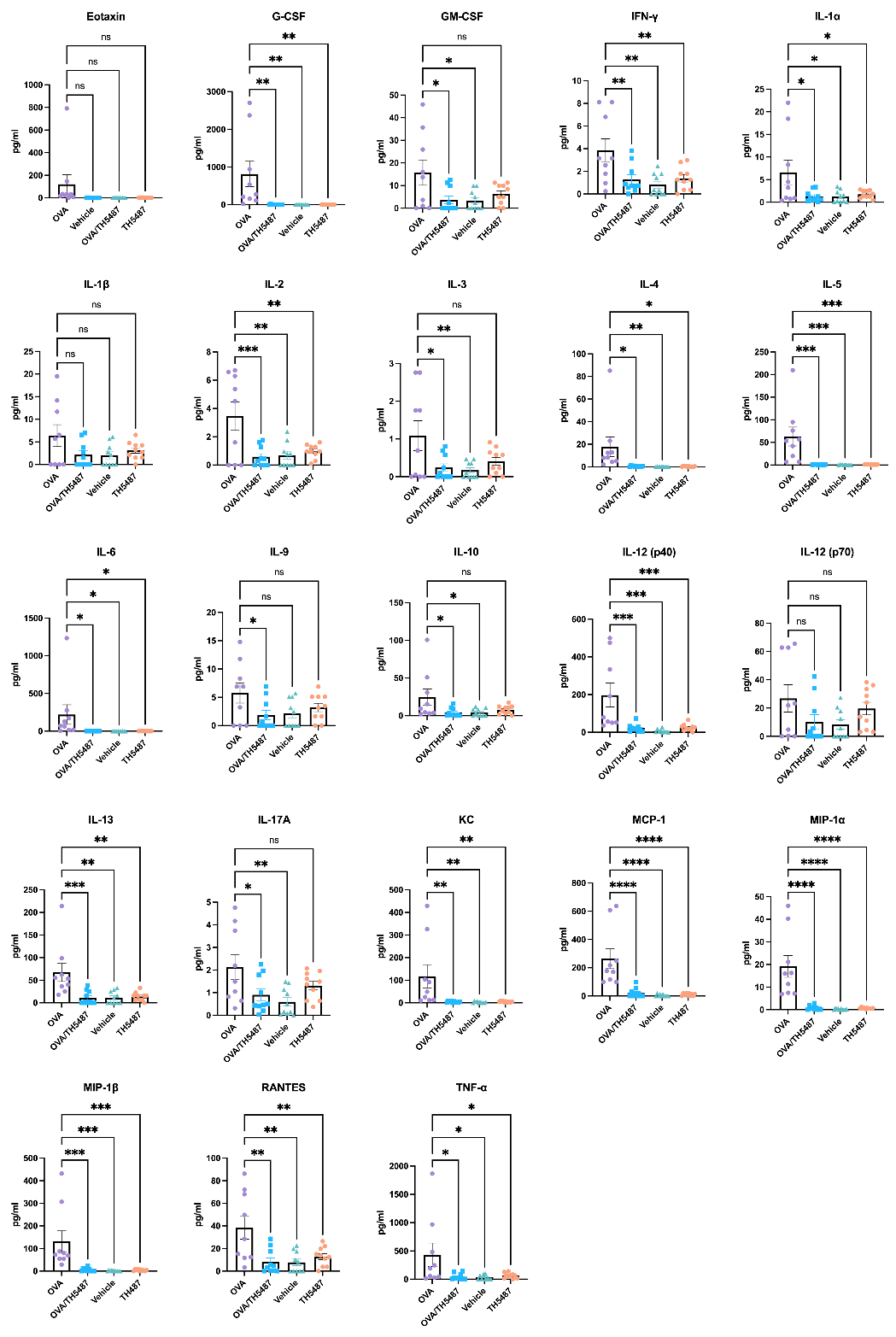


**Supplementary figure 7:** Individual graphs of all cytokines measured in BALF. Statistical comparison between groups was performed using a one-way ANOVA with Dunnett’s post-hoc test (*****P*<0.0001, ****P*<0.001, ***P*<0.01, **P*<0.05).

**Supplementary figure 8:** Individual graphs of all cytokines measured in plasma. Statistical comparison between groups was performed using a one-way ANOVA with Dunnett’s post-hoc test (*****P*<0.0001, ****P*<0.001, ***P*<0.01, **P*<0.05).


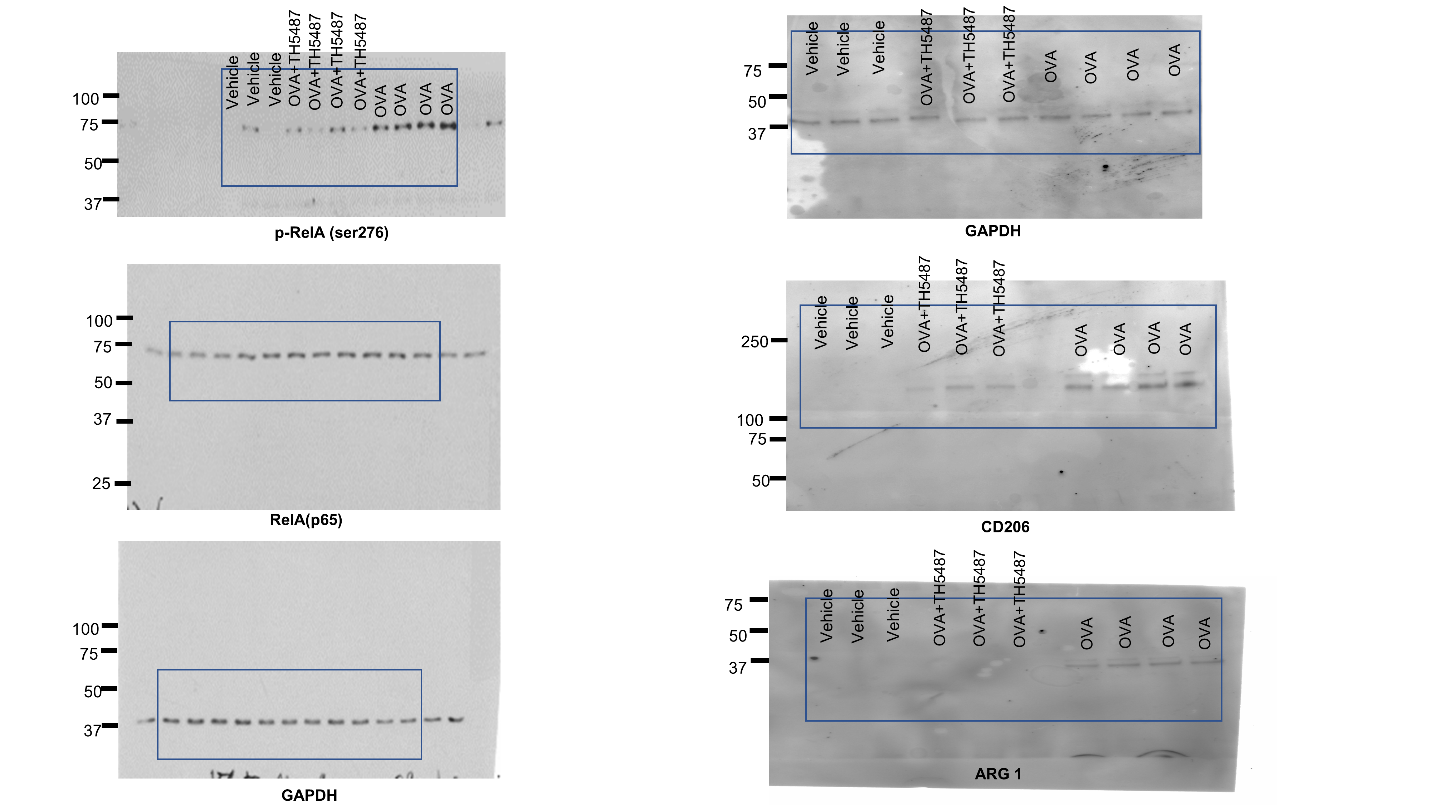


**Supplementary Figure 9: Representative Western blots conducted on murine lung tissue from OVA-challenged mouse lung experiments.** GAPDH was utilized as a control for all murine lung sample blots. P-RelA (ser276; MW: 67 kDa); RelA (p65; MW: 70 kDa), GAPDH (MW: 38 kDa); CD206 (MW:170 kDa); Arginase 1 (ARG 1; MW: 36 kDa).

**Supplementary Table 1:** Probes used for Real-time PCR analysis.

| **Gene** | **Probe** |
| --- | --- |
| *Sdha* | Mm01352366_m1 |
| *Arg1* | Mm00475988_m1 |
| *Mrc1* | Mm01329359_m1 |
